# Modeling intracranial electrodes. A simulation platform for the evaluation of localization algorithms

**DOI:** 10.3389/fninf.2022.788685

**Published:** 2022-10-06

**Authors:** Alejandro O. Blenkmann, Anne-Kristin Solbakk, Jugoslav Ivanovic, Pål Gunnar Larsson, Robert T. Knight, Tor Endestad

**Affiliations:** ^1^Department of Psychology, University of Oslo, Oslo, Norway; ^2^RITMO Centre for Interdisciplinary Studies in Rhythm, Time and Motion, University of Oslo, Oslo, Norway; ^3^Department of Neurosurgery, Oslo University Hospital, Oslo, Norway; ^4^Department of Neuropsychology, Helgeland Hospital, Mosjøen, Norway; ^5^Department of Psychology, Helen Wills Neuroscience Institute, University of California, Berkeley, Berkeley, CA, United States

**Keywords:** SEEG, ECoG, iEEG, intracranial electrodes, depth electrodes, subcortical grids, subdural grids

## Abstract

**Introduction:**

Intracranial electrodes are implanted in patients with drug-resistant epilepsy as part of their pre-surgical evaluation. This allows the investigation of normal and pathological brain functions with excellent spatial and temporal resolution. The spatial resolution relies on methods that precisely localize the implanted electrodes in the cerebral cortex, which is critical for drawing valid inferences about the anatomical localization of brain function. Multiple methods have been developed to localize the electrodes, mainly relying on pre-implantation MRI and post-implantation computer tomography (CT) images. However, they are hard to validate because there is no ground truth data to test them and there is no standard approach to systematically quantify their performance. In other words, their validation lacks standardization. Our work aimed to model intracranial electrode arrays and simulate realistic implantation scenarios, thereby providing localization algorithms with new ways to evaluate and optimize their performance.

**Results:**

We implemented novel methods to model the coordinates of implanted grids, strips, and depth electrodes, as well as the CT artifacts produced by these. We successfully modeled realistic implantation scenarios, including different sizes, inter-electrode distances, and brain areas. In total, ∼3,300 grids and strips were fitted over the brain surface, and ∼850 depth electrode arrays penetrating the cortical tissue were modeled. Realistic CT artifacts were simulated at the electrode locations under 12 different noise levels. Altogether, ∼50,000 thresholded CT artifact arrays were simulated in these scenarios, and validated with real data from 17 patients regarding the coordinates’ spatial deformation, and the CT artifacts’ shape, intensity distribution, and noise level. Finally, we provide an example of how the simulation platform is used to characterize the performance of two cluster-based localization methods.

**Conclusion:**

We successfully developed the first platform to model implanted intracranial grids, strips, and depth electrodes and realistically simulate thresholded CT artifacts and their noise. These methods provide a basis for developing more complex models, while simulations allow systematic evaluation of the performance of electrode localization techniques. The methods described in this article, and the results obtained from the simulations, are freely available via open repositories. A graphical user interface implementation is also accessible via the open-source iElectrodes toolbox.

## Introduction

Intracranial subdural grids and depth electrodes are implanted in patients with drug-resistant epilepsy as part of their pre-surgical evaluation. Electrophysiological and neuroanatomical data are used to delineate the seizure onset zone and functional areas that will guide respective surgery (Rosenow and Lüders, 2001). Intracranial electroencephalography (iEEG) recordings provide insights into human brain electrophysiology and functional mapping with unparalleled spatial and temporal resolution, offering both clinical and research applications. Knowing the exact location of electrodes in relation to the individual cortical or subcortical anatomy is a prerequisite for a complete understanding of the electrophysiological data; leading to a precise resection of the epileptic foci and the anatomical localization of specific brain functions (Lachaux et al., 2003; Parvizi and Kastner, 2017; Frauscher et al., 2018; Stolk et al., 2018).

One of the most common approaches to define electrode coordinates is to localize their artifacts in post-implantation computer tomography (CT) images co-registered to pre-implantation magnetic resonance imaging (MRI) scans. Typically, electrode CT artifacts are extracted by thresholding CT images, and then electrode coordinates are manually or semi-automatically computed from these (Blenkmann et al., 2017; Stolk et al., 2018; Li et al., 2019; Centracchio et al., 2021; Rockhill et al., 2022). In both cases, errors associated with the procedure are rarely quantified. It is well known that other CT artifacts than the ones of interest (e.g., connection cables or clips), adjacent electrodes, overlapping grids or strips, and noise or low image resolution make precise localization problematic (Brang et al., 2016; LaPlante et al., 2016; Narizzano et al., 2017).

Other localization approaches are based on the use of post-implantation MRI and CT images (LaViolette et al., 2011b; Hinds et al., 2018), clinical neuronavigational data (Gupta et al., 2014), or electrophysiological data (Branco et al., 2018b). However, these data might not be available in some clinical setups.

Over the last few years, the spatial resolution of grids and depth electrodes has reached inter-electrode distances (IED) of 2–3 mm, and will likely improve even more (Gupta et al., 2014; Chang, 2015; Martin et al., 2018). Given their size and the low signal-to-noise ratio (SNR) of images, high-density (HD) arrays are harder to localize with currently available methods [see novel attempts in Hamilton et al. (2017), Narizzano et al. (2017), Branco et al. (2018a), and Erhardt et al. (2020)].

Although methods to localize CT artifacts and co-localize them to pre-implantation MRI are common, there is no reliable gold standard to quantify their precision and robustness against noise. Methods are usually validated with different datasets, where noise is not quantified. This lack of standardization in the validation makes the comparison of methods imprecise and leaves unanswered questions: what is the localization error associated with a particular SNR? Which localization method performs better for a particular case? These are important factors undermining the validity of claims regarding specific brain areas related to a function or pathology.

To aid in solving these problems, we propose a new platform to model realistic intracranial electrode implantation scenarios and their respective CT artifacts, enabling systematic quantification of errors in electrode localization algorithms.

In the next sections, we will introduce methods for fitting subdural grid and strip electrode models onto the smooth hull surface, and depth electrode array models targeting subcortical sites. We then describe the modeling of thresholded CT artifacts and their noise. Thereafter, we report the simulation results and their validation with real data. Moreover, we provide two examples: (i) performance evaluation of localization methods, and (ii) modeling implantations on a patient’s native anatomy. Finally, we discuss the results in the context of existing electrode localization methods, the limitations of our approach, and future challenges.

The methods and results presented in this article are publicly available (except for individual patient data).

## Materials and methods

**FIGURE 1:**
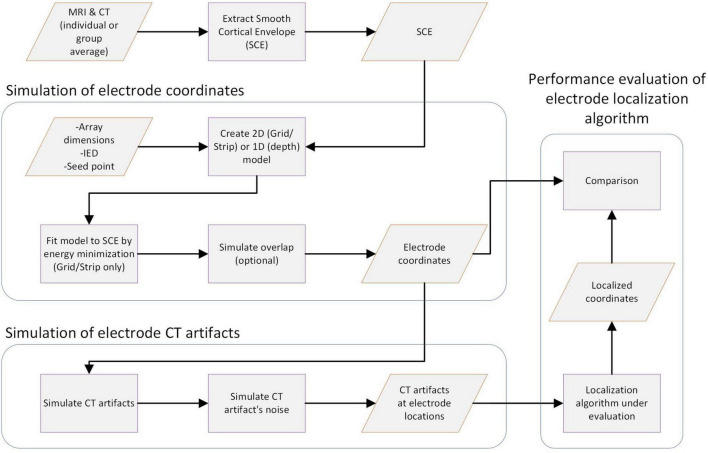
Workflow for simulating electrode CT artifacts and evaluating localization algorithms. The main steps of the workflow are shown in the diagram. First, a Smooth Cortical Envelope (SCE) is obtained from an individual or average atlas image. Electrode arrays are modeled according to design parameters and fitted over the SCE (grids) or placed within the brain tissue (depths) to obtain the electrode coordinates. Then, CT artifacts are simulated at each electrode coordinate, considering the electrode type and spatial orientation. Subsequently, noise is added to simulate different realistic scenarios. CT artifacts are then fed into the localization algorithm under evaluation. Finally, localized coordinates are compared with the ground truth electrode coordinates. Knowing the true electrode coordinates allows a correct estimation of the localization error and its sensitivity to different noise levels. Moreover, the results can be easily replicated. Orange parallelograms represent data, and purple squares represent processes.

The proposed platform allows the systematic evaluation of intracranial electrode localization algorithms. The workflow consists of several steps. Concisely, they can be grouped into pre-processing of images to obtain a Smooth Cortical Envelope (SCE), the simulation of electrode coordinates, the simulation of CT artifacts, and the performance evaluation. Figure 1 shows the main steps, and the following subsections describe them in detail.

Unless otherwise specified, the methods were implemented in Matlab R2019 (The MathWorks Inc., USA) and iElectrodes toolbox for Matlab (Blenkmann et al., 2017). The most relevant functions needed to perform the simulations are mentioned along the method’s subsections.

In general, median values were used to describe the central tendencies of the variables of interest since most distributions were not normal. Their corresponding CIs were obtained by bootstrapping.

### Smooth cortical envelope surface and seed points

The implantation of real grids and strips onto different cortical areas introduces variations in how these arrays bend to follow the brain curvature. To reproduce realistic scenarios, we simulated grids implanted over 3D cortical surfaces. High-resolution pial surfaces extracted from the MNI atlas were used as a starting point (or individual structural MRI images for simulations in native space; see subsection “Example II: Interactive modeling of implanted electrodes in patient’s native brain space” for details). In our processing pipeline, we used Freesurfer software (Dale et al., 1999), and individual cortical parcellation images were obtained using the Destrieux atlas (Destrieux et al., 2010), but other atlases or software could also be used. We then computed an SCE surface for each cerebral hemisphere by enclosing the corresponding pial surface with a 30 mm radius sphere. A mesh smoothing was applied to remove small local protuberances (low-pass spatial filter, 100 iterations, alpha weight = 0.5, Iso2Mesh toolbox, Fang and Boas, 2009). Supplementary Figure 1 shows an example of the pial surface and SCE computed for an individual patient’s brain. The procedure is implemented in the *buildSCE.m* function.

We visually selected 57 seed points over the SCE surface and used them as reference points to model grids, strips, and depth electrode arrays. We computed the local curvature of a smoothed version of the SCE (Rusinkiewicz, 2004). Seed points were visually selected on regions where the local curvature was relatively *“Low,” “Medium,”* or *“High”* within the range of curvature values. We modeled different electrode arrays depending on the location and the local curvature of the SCE surface.

### Modeling grid and strip electrode coordinates

**FIGURE 2:**
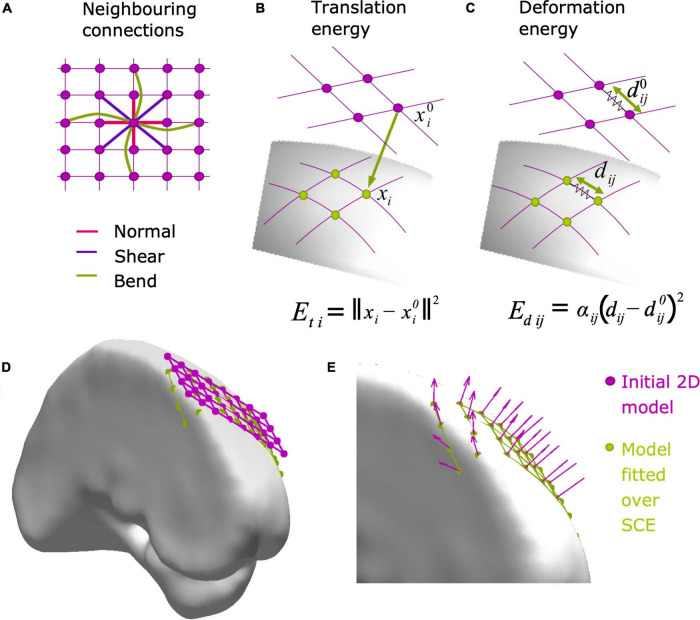
Modeling grid and strip electrode coordinates over the SCE surface. Electrode arrays are initially modeled as 2D (grids) or 1D (strips) objects, and then fitted to the SCE surface by minimizing the translation (*E*_t_) and deformation (*E*_d_) energies (Equation 1). **(A)** First, diagonal, and second neighboring connections are used to compute the deformation energy *E*_d_, accounting for normal, shear, and bending, respectively. **(B)** The translation energy *E*_ti_ associated with electrode i is proportional to the distance between the initial (*x*^0^_i_) and the final location (*x*_i_) over the SCE surface. **(C)** The deformation energy *E*_dij_ associated with neighboring electrodes i and j is proportional to the amount of deformation between the original (*d*^0^_ij_) and final distance (*d*_ij_). Equation details are explained in Subsection “Modeling grid and strip electrode coordinates”. **(D)** Example of a 2D grid model (4 × 8, 10 mm IED) before (pink) and after (green) being fitted to the SCE surface on the left frontal lobe. **(E)** Zoom-in detail showing the normal vectors at each electrode location. Normal vectors are subsequently used to orient the simulated CT artifacts. IED, inter-electrode distance; SCE, smooth cortical envelope.
(2) The 2D model is fitted over the SCE surface using an energy minimization algorithm (Figure 2D, Dykstra et al., 2012; Trotta et al., 2018) implemented in the function *projection2mesh.m*. The energy function

The following steps are performed for modeling subdural grids and strips:

(1) A 2D flat model of the array is generated and placed tangentially to the SCE in a given “seed” point (e.g., the purple grid in Figure 2D) using the function *tangentialGrid.m*. For strip cases, the number of rows is set to three to avoid unrealistic geometrical deformations (e.g., undulating shapes).

(1) $$E=E_{t}+KE_{d}$$

is minimized by varying the electrode coordinates to the final position *x*_*i*_, where *E*_*t*_ is the translation energy, *E*_*d*_ is the deformation energy, and *K* is a constant value. The energy minimization is constrained to the electrode coordinates *x*_*i*_ being closely located over the SCE surface, i.e., ∀*i*, ||*x*_*i*_−*s*_*i*_|| ≤ ϵ, where *s*_*i*_ is the closest point in the SCE surface to electrode *i*, and ε is a tolerance distance.

*E*_*t*_ (Figure 2B) represents the energy needed to translate the electrode coordinates from the original position of each electrode $x_{i}^{0}$ to the final position *x*_*i*_ on the SCE surface:


(2) $$E_{t}=\sum_{i=1}^{N}{||x_{i}-x_{i}^{0}||}^{2}$$

where *N* is the number of electrodes.

*E*_*d*_ (Figure 2C) represents the energy required to change the inter-electrode euclidean distance between neighbors from the original to the final location. It is defined as


(3) $$E_{d}=\sum_{i=1}^{N}\sum_{j=i+1}^{N}\alpha_{ij}{\left(d_{ij}-d_{ij}^{0}\right)}^{2}$$

where *d*_*ij*_ is the distance between electrodes, $d_{ij}^{0}$ is the initial distance between contacts, and *α_*ij*_* takes values of 1 or 0 if electrodes *i* and *j* are neighbors or not, respectively. To control for normal, bending, and shear deformations, first, second, and diagonal (grids only) neighbors are considered, as shown in Figure 2A (Trotta et al., 2018). *E*_*d*_ is typically interpreted as the deformation of “springs” connecting the electrodes (Dykstra et al., 2012).

(3) A normal vector at each electrode coordinate is computed. We select the coordinates from each electrode of interest and the nearest neighbors and compute a principal component analysis (PCA) of these points. The normal vector is the one associated with the smallest component (Figure 2E).
(4) For strips, only the middle row coordinates are kept from the three rows, while the lateral ones are discarded.

We simulated electrode arrays of multiple dimensions representing models frequently available in the market by different manufacturers. These cover various combinations of size and IED, including grids of 2 × 4, 4 × 4, 4 × 8, 8 × 8, 8 × 16, and 16 × 16 contacts, strips of 1 × 4, 1 × 6, and 1 × 8 contacts. Arrays were simulated at the 57 seed points in the standardized MNI SCE surface with different local curvatures (Supplementary Figure 2).

Grid and strip implantation scenarios were simulated several times at each seed point, rotating the grids at angles multiple of 30 degrees around the center. The location and the local curvature of the SCE surface surrounding the seed points determined which arrays were simulated. Large grids were simulated over *Low* curvature regions, whereas medium and small size grids were simulated over *Medium* and *High* curvature areas. For example, over the lateral fronto-temporo-parietal cortex, it is realistic to simulate an 8 × 8, 10 mm IED grid, but unrealistic (in usual clinical settings) to simulate the implantation of such a big grid over the frontal pole.

For the fitting of grid and strips onto the SCE surface, coefficient *K* was set to 1,000, and the tolerance distance ε = 0.1 mm for all arrays, except for the 16 × 16 grid cases where *K* = 100 and ε = 0.5 mm (Equation 1).

Deformations are needed to fit a grid or strip (originally plane objects) over the brain surface (a curved object). When localizing grids, we typically observe that these deformations are small. Therefore, we measured the IED after fitting grids and strips to the SCE surfaces and discarded simulations if one or more neighboring electrodes had IED variations over 5% from the original values. A simple example of the simulation is implemented in the *simulateArraySCE.m* function.

### Modeling depth electrode coordinates

In clinical practice, the implantation of depth electrodes is defined by two points, and therefore a unique trajectory connecting them. The points are typically defined as an “entry point” on the cortical surface and a “target point” at the deepest brain location reached. We will adopt this nomenclature throughout this paper.

It is common practice to use trajectories orthogonal to the skull surface to avoid sliding of the drill and minimize bone damage during surgery. Therefore, given an entry point on the SCE surface, we define the array trajectory vector orthogonal to this surface. Two strategies are applied to distribute the electrodes along the trajectory uniformly:

(1) If the array is shorter than the amount of tissue intersected by the trajectory, the target point is defined as a random point within the trajectory while keeping all contacts inside the brain.
(2) If the array is longer than the intersected tissue, the target point is defined as the deepest electrode point in the trajectory within the brain tissue (enclosed by the SCE surface). Contacts outside the brain tissue are removed.

As an optional step, a curvature deformation can be applied within a random orientation 2D plane containing the principal axes of the array. Using the symmetric Lanczos window, an arc-shaped electrode array is obtained. A simple example of the simulations is implemented in *simulateDepthArray.m* function.

We selected 33 entry points over the MNI SCE from the previous set of 57 seed points, restricted to locations where the implantation of depth electrodes were realistic. At each point, we simulated the implantation of depth electrodes as combinations of 4, 8, 10, 12, 15, and 18 contacts; 3, 5, and 10 mm of IED; and linear or curved deformation (maximum deformation of 1% of the total length).

### Modeling overlapping grids and strips coordinates

**FIGURE 3:**
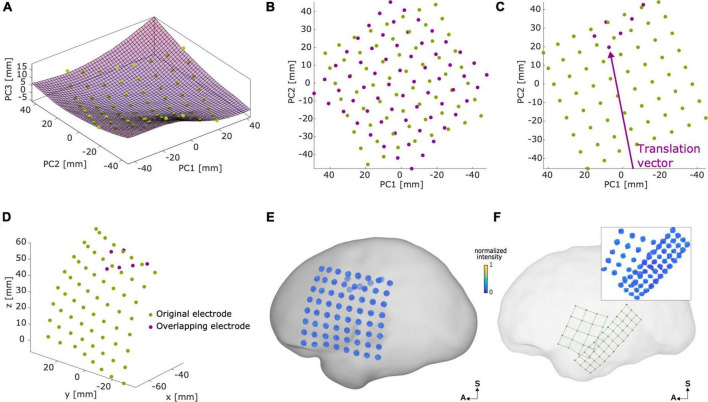
Modeling overlapping grids. Example of an overlapping grid in an 8 × 8, 10 mm, IED case. **(A)** Original electrode coordinates (green) were transformed into the principal component space, where a surface (*S*_fit_) was fitted to the coordinates. **(B)** In a 2D space defined by PC1 and PC2, the overlapping grid (magenta) was defined with a random orientation. **(C)** The overlapping grid was translated (in the arrow direction) until the desired number of overlapping electrodes was achieved. **(D)** Coordinates were back-projected to the original 3D space surface. **(E)** Voxel artifacts were simulated in the new overlapping grid electrode coordinates, overlaying the SCE surface. Voxel artifacts’ orientations were interpolated from close-by electrodes of the original array. **(F)** Example of real overlapping grids (5 × 4, 10 mm IED and 4 × 12, 6 mm IED) overlaying the left SCE surface. The inset shows the corresponding thresholded CT artifact voxels. Plots **(B,C)** show the PC1 axes direction inverted for illustrative purposes. IED, inter-electrode distance; SCE, smooth cortical envelope; A, anterior; S, superior.

Overlapping grids or strips are obstacles for methods that aim to detect intracranial electrodes automatically (e.g., Figure 3F). We developed a method to realistically simulate these cases (Figure 3E), implemented in the function *simulateOverlaps.m.* Briefly, it consists of:

(1) A surface is fitted to the background array (Figure 3A).
(2) The coordinates are projected to a 2D principal component space (PCA) where the overlapping array is initially positioned (Figure 3B).
(3) The overlapping array is displaced in a random direction until the desired amount of overlap is achieved (Figure 3C).
(4) The coordinates are back-projected to the initial surface (Figure 3D).

A more detailed explanation of the procedure can be found in Supplementary Section 3.

Grids and strips were simulated with a 10% overlap and no overlap. Overlapping grids were defined with a random orientation and translated in a random direction. In principle, grids and strips of any size and IED can be overlapped. However, for the sake of simplicity, we used arrays with the same dimensions.

### Modeling electrode computer tomography artifacts and their noise

Grid and depth arrays have a flat disc and cylinder-shaped metallic contacts, respectively. Their corresponding thresholded CT artifacts usually show ellipsoidal shapes, and their voxels’ intensity typically decreases with their distance to the center of the electrode (Blenkmann et al., 2017; Stolk et al., 2018). Therefore, we modeled the CT artifacts representing electrodes as ellipsoidal-shaped clusters of voxels of varying intensity. In a parsimonious approach, we defined the intensity as a linear function that declined with the scaled radial distance *r* of a given location (*x, y, z*) to the ellipsoid center as:


(4) Intensity(x,y,z)=1-r(x,y,z)

where


(5) $$r\left(x,y,z\right)=\sqrt{{\left(\frac{x}{a}\right)}^{2}+{\left(\frac{y}{b}\right)}^{2}+{\left(\frac{z}{c}\right)}^{2}}$$

and *a*, *b*, and *c* are the semi-axes of the ellipsoid model. Semi-axes length parameters were defined depending on electrode type, their inter-electrode distance, following manufacturers’ specified dimensions, and visually adjusted to match real CT artifacts’ observed size and shape. Specifically, we used *a* = 2.2, *b* = 2.2, and *c* = 1.5 for 10 and 5 mm IED grids, *a* = 1.1, *b* = 1.1, and *c* = 1 for 3 mm IED grids, *a* = 1.25, *b* = 1.25, and *c* = 1.75 for 10 and 5 mm depth electrode arrays, and *a* = 1.1, *b* = 1.1, and *c* = 1.5 for 3 mm IED depth electrode arrays.

The function *simulateVoxels.m* was used to simulate the clusters of voxels for each CT artifact. First, voxel coordinates were generated by using a 0.5 mm resolution 3D square lattice grid of 10 mm × 10 mm × 10 mm around each electrode to represent the effect of discrete sampling in CT images. All sampling lattice grids had a common random origin and orientation mimicking the random location and orientation of the patient’s head in the image reference system. Then, we centered the reference coordinate system at the electrode’s coordinate. We rotated the reference system to align its *z*-axis to the electrode’s specific normal vector, i.e., the surface’s orthogonal vector for grids and strips (Figure 2E) or the trajectory vector for depths. Finally, we computed the intensity of each voxel using Equation 4.

The localization of intracranial electrodes is typically sensitive to the signal-to-noise ratio of the processed CT images. To model the noise in the CT artifacts, we added spatially correlated and uncorrelated noise to each voxel’s intensity using the function *addnoise2electrodes.m* (Britten et al., 2004; Gravel et al., 2004). Spatially correlated noise was computed by convolving uncorrelated noise from a $N\left(0,\sigma_{corr}^{2}\right)$ distribution with a kernel provided by Britten et al. (2004). Uncorrelated noise values were drawn from a $N\left(0,\sigma_{uncorr}^{2}\right)$ distribution. Therefore, the total noise variance was $\sigma_{total}^{2}=\sigma_{corr}^{2}+\sigma_{uncorr}^{2}$. The two noise sources contributed differently to the deformations observed in the CT artifacts.

Finally, voxels with intensity values below zero were removed after adding noise.

We simulated the CT artifacts at each electrode location and manipulated the noise levels affecting these. Twelve noise levels were simulated for each scenario, where $\sigma_{total}^{2}$ was logarithmically distributed between 0.2 and 2.2 (from now on denoted as noise levels 1–12). The ratio between correlated and uncorrelated noise variances was set to 20:1, a heuristically determined ratio obtained by observing the shape of CT artifacts. The individual impact of these two noise sources was clear at high noise levels. Higher ratios of uncorrelated noise left many “disconnected” voxels surrounding the CT artifacts, whereas higher ratios of correlated noise introduced unrealistically deformed shapes.

### Validation

#### Patients

To validate our modeling platform, we compared our simulations with real data. We analyzed MRI and CT images from 17 adult patients with drug-resistant epilepsy who underwent iEEG recording as part of the pre-surgical evaluation for respective surgery.

Three patients were implanted with only subdural grids, seven with subdural grids and depth electrodes, and seven with only depth electrodes. IED ranged from 3 to 10 mm for both grids and depth arrays. Patients implanted with depth electrode arrays had on average 69 depth contacts (range 8–172 contacts per patient, total of 1,068 contacts, DIXI Medical, France, Ad-Tech Medical Instrument Corporation, USA, or PMT Corp., USA). Patients implanted with subdural grids had on average 146 subdural contacts (range 29–306 contacts per patient, total of 1,656 contacts, Ad-Tech Medical Instrument Corporation, USA, or PMT Corp., USA).

#### Electrode localization in real cases

We followed a routine procedure to localize intracranial electrodes (Stolk et al., 2018; Blenkmann et al., 2019). Pre-implantation T1-weighted MRI images were processed using the FreeSurfer standard pipeline (Dale et al., 1999), where individual brain segmentation images, pial surfaces, and cortical parcellation images (Destrieux atlas) were obtained (Destrieux et al., 2010). Post-implantation CT images were co-registered to the pre-implantation MRI using SPM 12 software (Studholme et al., 1999). Realigned MRI and CT images were resampled to 0.5 mm × 0.5 mm × 0.5 mm resolution. CT images were thresholded to visualize the clusters of high-intensity voxels (also known as CT artifacts). Threshold values were visually defined to identify clusters of voxels representing individual electrodes following the procedure described in Blenkmann et al. (2017).

Each cluster of high-intensity voxels was extracted using the *k*-medoids clustering algorithm (as implemented in iElectrodes toolbox). Then, the weighted center coordinates of each cluster were computed. In noisy situations, the center of each cluster was visually identified instead of using automatic clustering.

#### Validation of electrode coordinates

To validate the modeling of electrode array coordinates, we visually inspected the resulting coordinates. Grid and strip coordinates were expected to follow the SCE surface, while only minimally deforming the IED. Moreover, we described deformations in the distance between their first, second, and diagonal neighbors. To integrate the impact of these deformations in the framework of electrode localizations, we qualitatively compared these deformations with those obtained from manually localized real arrays of 10 mm IED. We anticipated simulation deformations to be smaller than the localized ones, since the latter includes array deformations and localization errors.

In addition, we evaluated the deformations of depth arrays. First, array lengths were normalized to allow their comparison. Then, we defined the main axis connecting the first and last contact. Finally, we measured the shortest distance between each contact and the axis.

#### Validation of computer tomography artifacts

To validate our model of electrode CT artifacts, we compared the characteristics of these synthetic artifacts with those of real artifacts. Noise characterization was particularly relevant since it can affect the accuracy of localization algorithms. We applied an information theory measure, entropy, to delineate the noise level of simulated and real data. With this aim, we first computed the principal axis of each electrode. For grids, we computed the orthogonal direction at each electrode given its closest neighbors, and for depth electrodes, we computed the principal axis as the direction connecting the first and last electrodes of the array. We aligned the principal axes to the *z*-axis and positioned their centers at the coordinate system’s origin. Then, the 2-dimensional intensity-radius histograms were computed, showing the bivariate distribution of voxels in terms of intensity (on the *y*-axis) and radial distance to the center (on the *x*-axis). Histograms were computed in 0.25 mm bins between 0 and 5 for the radial distance, and 0.05 bin size for the normalized intensity values between 0 and 1, while excluding intensity values below percentile 2.5 and above percentile 97.5. Afterward, we computed the pairwise 2D cross-correlation matrices between all histograms within an array. A cross-correlation matrix indicates the degree of similarity between two intensity-radius histograms as a function of the displacement of one relative to the other. Sharp cross-correlations are indicative of similar histograms, whereas smother (more distributed) cross-correlations indicate less consistency between the histograms. In our case, we expected the noise to differently affect each CT artifact within an array. Therefore, this should be reflected in smother cross-correlations of their corresponding 2D histograms when noise levels are high, and sharp and peakier cross-correlations when noise levels are low. Finally, we estimated the array’s noise by measuring the entropy of these cross-correlation images and computing their average. Entropy values represent the amount of disorder present in the cross-correlation matrices. Entropy has been previously used to estimate the focus of images, by evaluating the uniformity of the 2D spectral decomposition of images (Bove, 1993; Kristan and Pernu, 2004). Similarly, in our case, entropy values will increase as the cross-correlation images get close to uniformly distributed (e.g., smoother images) and decrease as they get farther from uniform (e.g., peakier images). Therefore, low entropy values indicate more consistent, i.e., less noisy, artifacts within an array, and vice versa. Entropy values were sensible to the size of the electrodes. Therefore, entropy values were normalized by subtracting each electrode size group’s mean value.

To evaluate the similarity between simulation and real data arrays, we computed the average correlation across their electrode histograms (normalized by the mean correlation within electrodes of the real array). If our model is able to capture the effect of noise in real data, real electrodes with high noise levels should correlate better with high noise level simulations. To test this hypothesis, we used a linear model to evaluate the relationship between the estimated noise level in real electrodes and the noise level of the highest correlating (i.e., best fitting) simulation.

Grids with less than twelve electrodes were discarded. These grids have a high proportion of edge-located electrodes which can lead to less accurate estimations of the orthogonal vector and affect the following analysis.

The spatial organization of electrodes in strips precludes the computation of orthogonal vectors and, therefore strips were not included in the current analysis. However, strip electrodes are usually the same size as grid electrodes, and we assume that the conclusions drawn from the analysis of grids also apply to strips.

### Example I: Evaluation of cluster-based localization methods

We demonstrate the applicability of our modeling platform by evaluating the performance of two localization approaches implemented in v1.000 and v1.010 of the iElectrodes toolbox. These are based on the *k-means* and *k-medoids* clustering algorithms, respectively (Hartigan and Wong, 1979; Kaufman and Rousseeuw, 1990). Before running the localization scripts, artifacts were thresholded between 0.25 and 0.6 depending on the noise level.

Localized coordinates were contrasted with simulated coordinates to evaluate their accuracy under several noise levels. The methods were compared with the Wilcoxon signed-rank tests, and effect sizes were approximated from their estimated *z*-scores (Pallant, 2007).

The function *localizeArray.m* and dependencies provide an exemplary code for testing localization algorithms. The script can be easily modified for comparison of other localization algorithms.

### Example II: Interactive modeling of implanted electrodes in patient’s native brain space

To demonstrate the algorithm’s usefulness in more realistic scenarios, we applied our methods in a patient’s native brain space. We processed pre-implantation MRI and post-implantation CT images from an adult patient with drug-resistant epilepsy, a potential candidate for respective surgery, as described in Section “Patients.” The SCE surfaces for each hemisphere were computed following the steps described in Section “Smooth cortical envelope surface and seed points.”

We simulated grids, strips, and depth electrodes over multiple center points (seeds) and orientations using the interactive Graphical User Interface (GUI) provided by the iElectrodes toolbox (Blenkmann et al., 2017). For grids and strips, the 2D flat model arrays were first manually translated and rotated over the 3D SCE surface (Figure 2D). Once the desired location was reached, they were fitted to the SCE surface. Depth electrodes were defined by visually setting the target and entry points in the 2D views.

## Results

In the following subsections, we will present the simulation results, their validation with real data, and examples.

### Simulation and validation of electrode array coordinates

A total of 3,646 scenarios for grids and strips were simulated, and in 3,321 instances the arrays were successfully placed over the SCE surface. In 9% of the cases, the IED deformations between at least one pair of contacts exceeded the 5% threshold and were discarded.

For depth electrodes, the procedure resulted in 858 simulation scenarios for depth electrodes within the MNI brain.

**FIGURE 4:**
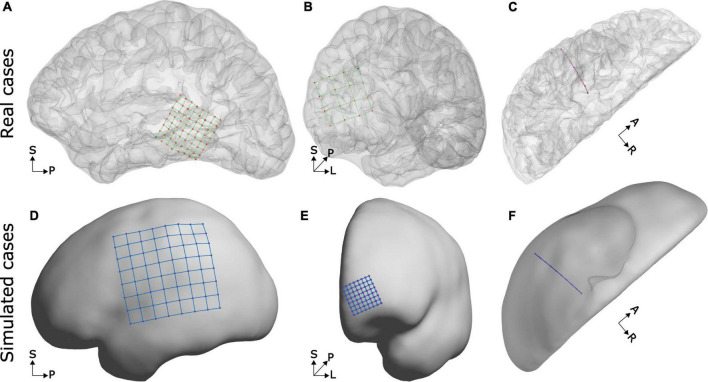
Examples of real and simulated electrode coordinates. Observe that in real and simulated cases, the grid coordinates follow the brain curvature and that depth electrode trajectories are slightly bent. The top row shows real electrode coordinates from an 8 × 8, 3 mm IED array over the lateral temporal cortex **(A)**, a 4 × 4, 10 mm IED array over the frontal pole **(B)**, and an 8 contacts, 5 mm IED, depth electrode array into the temporal lobe **(C)**. The bottom row shows simulated electrode arrays from an 8 × 8, 10 mm IED grid over the lateral fronto-temporo-parietal cortices **(D)**, an 8 × 8, 3 mm IED high-density array over the frontal pole **(E)**, and a 10 contacts depth electrode array into the temporal lobe **(F)**. SCE and pial surfaces are depicted semi-transparently in the top plots to enhance visualization.

We first validated our results by visual observation. Grid and strip electrode coordinates closely followed the SCE surface and showed an almost uniform spatial distribution. Changes in the IED were difficult to detect. For example, Figures 4A,B,D,E show simulated and real localized grid coordinates. Another important aspect was the simulation of overlaps, comparable to those observed in real situations (Figures 3E,F). Besides, the curved trajectories of simulated depth arrays mimicked those of real cases (Figures 4C,F).

The implantation of subdural electrode arrays often introduces a brain shift, and this causes post-implantation CT images to capture the electrode artifacts “buried” under the pial or SCE surface reconstructed from pre-implantation MRIs. This deformation of the brain surface is not modeled in our current implementation.

**FIGURE 5:**
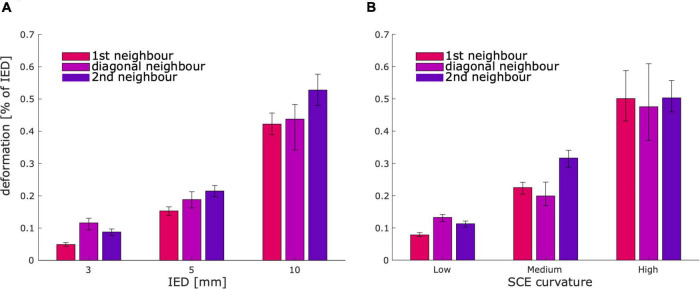
Deformation between contacts for simulated grids and strips. Bar plots show that the median deformation between contacts increases with the IED **(A)**, and the SCE curvature **(B)**. As a reference, the observed deformations in real cases were an order of magnitude larger (Supplementary Figure 3A). Error bars denote 95% CI of the median. SCE, smooth cortical envelope; IED, inter-electrode distance.

To evaluate the quality of the simulated grid and strip coordinates, we quantified the deformations introduced by projecting them onto the SCE. We measured the distance between 1st, diagonal, and 2nd neighboring contacts, normalized in each case by the IED. The median deformations were 0.174, 0.169, and 0.243% of the IED, respectively. The median deformation increased for higher values of IED and SCE curvature (Figures 5A,B). As an upper bound reference, we measured the IED deformations from CT localized real grids (13 arrays, 484 electrodes). These deformations were one order of magnitude larger than the simulated ones (Supplementary Figure 3A).

To better quantify the effect of curvature and the number of electrodes on the simulations, we measured the distance between the fitted electrodes and the SCE. Although these values were constrained in the fitting procedure, some variability was observed. The electrodes’ median distance to the SCE decreased with the number of contacts and increased with the SCE curvature (Supplementary Figure 4). Overall, the median distance of electrodes to the SCE was 0.012 mm.

To validate the bending model of depth arrays, we measured deformations in real cases. Across real arrays, the maximum deformation (i.e., the maximum normalized distance to the main axis) was 0.82%, 95% CI [0.72, 1.01], comparable to the 1% maximum deformation implemented in our approach. Supplementary Figure 3B shows the bending profile over distance and the Lanczos function used in the simulations. Interestingly, the profile indicates that the maximum deformation is not centered, but closer to the deepest contact (normalized distance to target 0.37, 95% CI [0.33, 0.43]).

### Simulation and validation of electrode computer tomography artifacts

We simulated the CT artifacts at each electrode location and manipulated the noise levels affecting these. By using the normal orientation vector, the procedure generated grid and strip CT artifacts aligned with the brain’s surface curvature. Similarly, depth electrode artifacts were aligned with the arrays’ principal axes. Altogether, we produced ∼40,000 simulations of grids and strips and ∼10,000 depth electrode CT artifacts.

**FIGURE 6:**
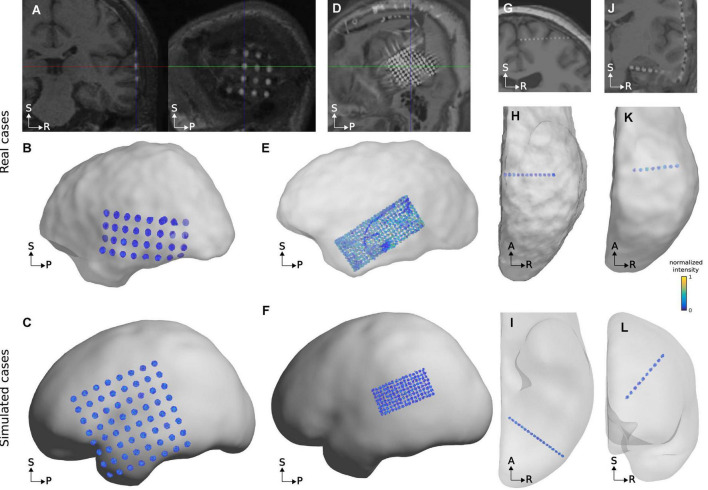
Examples of real and simulated electrode CT artifacts. Images on the top row **(A,D,G,J)** show electrode artifacts on CT images superimposed on co-registered MRIs from real cases. Plots on the middle row **(B,E,H,K)** show thresholded CT voxels corresponding to electrodes in relation to the SCE surface for the same cases as above. Plots on the bottom row **(C,F,I,L)** show simulations similar to the real examples above. Observe the similarity between the real and simulated CT artifacts. The examples selected have different noise levels to reflect some of the variability observed. The real grid case in **(A,B)**, and the correspondingly simulated grid **(C)** have relatively low noise, whereas the real case depicted in **(D,E)**, and simulation **(F)** show a higher noise level. The case depicted in **(E)** also shows a cable passing over the grid. Both real and simulated depth electrodes depicted in **(G–I)** have low noise levels, whereas cases shown in **(J–L)** have medium noise levels. Array details: **(A,B)** 4 × 8 grid, 10 mm IED, implanted over the temporal cortex. **(D,E)** 10 × 25 grid, 3 mm IED, implanted over the temporal cortex. **(G,H)** 12 electrodes depth array, 3.5 mm IED, implanted into the superior frontal cortex. **(J,K)** 8 electrodes depth array, 5 mm IED, implanted into the temporal cortex. **(C)** 8 × 8 grid, 10 mm IED, over the fronto-temporo-parietal cortex. **(F)** 4 × 8 grid, 3 mm IED, over the temporo-parietal cortex. **(I)** 18 electrodes depth array, 3 mm IED, implanted into the occipital cortex. **(L)** 10 electrodes depth array, 5 mm IED, implanted into the frontal cortex. For illustration purposes, the SCE surfaces are semi-transparent. IED, inter-electrode distance; SCE, smooth cortical envelope. A, anterior; P, posterior; R, right; S, superior.

**FIGURE 7:**
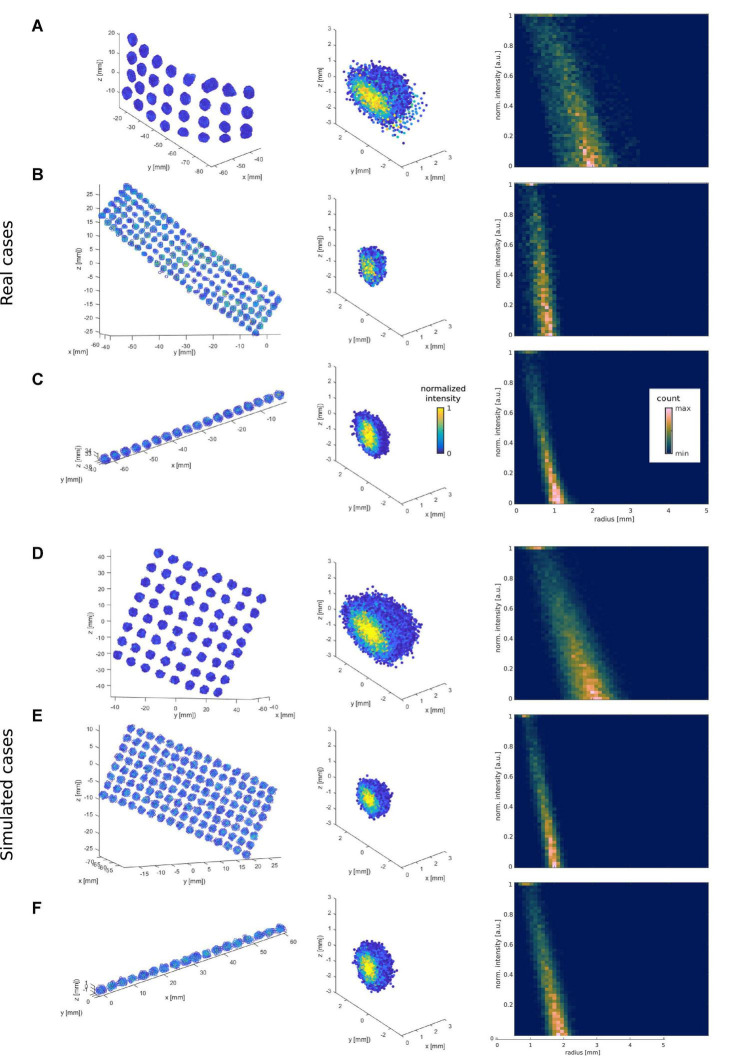
Examples of real and simulated electrode CT artifacts and their 2D intensity-radius histograms. The three top rows show examples of real CT artifacts obtained from a 10 mm IED grid **(A)**, a 3 mm IED high-density grid **(B)**, and a 3 mm IED depth electrode array **(C)**. The three bottom rows **(D–F)** show simulated cases with noise levels that best fit the real ones above (present the highest correlation). The left column plots show the spatial distribution of the voxels’ artifacts. The middle column shows the same artifacts after all individual electrodes were recentered at the origin (0,0,0), and their principal axes (orthogonally to the cortical surface for grids) or main axes (connecting outer electrodes for depths) aligned to the *z*-axis. For visualization purposes, only half of the electrode voxels are shown. The right column plots show their respective intensity vs. radius histogram. For illustrative purposes **(C,F)** middle and right column plots were obtained from 10 depth electrodes in the corresponding patient, or 10 simulated depth arrays, and not only from the individual arrays shown on the left column.

To validate our procedure, we first visually inspected the CT artifacts in a subset of 100 randomly selected simulations. Grid and depth arrays have flat disc and cylinder-shaped metallic contacts, respectively, whereas their CT artifacts usually have ellipsoidal shapes following these geometries (Figures 6, 7). Importantly, similar shapes were achieved by the simulations (Figures 6, 7).

Even under the effect of brain shift, we observed grids and strips electrodes in the CT images to be approximately parallel to the SCE obtained from pre-implantation MRIs (Figures 6B,E). Accordingly, simulated CT artifacts followed the SCE curvature (Figures 6C,F).

Moreover, noise levels gradually affected the shape of real artifacts. In general, larger noise levels hampered the visual identification of individual artifacts (e.g., Figures 6E,F). Importantly, similar effects were obtained in the simulations. Other noise sources, like cables or metallic clips, could also affect the visual identification, but were not simulated.

To further characterize electrode artifacts and their noise, we computed 2D histograms to capture the bivariate distribution of voxels in terms of intensity and radial distance to the center. In the real cases (15 grids across 11 patients, IED 3 and 10 mm; and 77 depth electrodes from 14 patients, IED 3 and 5 mm), we observed a tendency for intensity to decrease with radius (e.g., Figures 7A–C, right column), a pattern that was mimicked by the simulations (e.g., Figures 7D–F, right column). These 2D histograms allowed us to estimate the noise level that affected the intensity distribution of voxels in space. For this purpose, we computed the mean entropy of the 2D cross-correlation of histograms as a proxy for the noise level in each array. Supplementary Figure 5 shows examples of low and high noise arrays and their corresponding average 2D cross-correlation images.

As expected, incrementing the noise level in the simulations produced a systematic increase in their average entropy (Supplementary Figures 6C,D, 7C,D). We also observed that the entropy measure not only captured the noise level, but was also affected by the size of the CT artifacts, being higher for bigger artifacts in real and simulated cases. Therefore, entropy values were normalized by subtracting each electrode size group’s mean value to account for the artifact size’s effect on the entropy measure.

Entropy was also computed for real CT artifacts, allowing us to compare real and simulated CT artifacts in terms of noise content (Supplementary Figures 6A,B, 7A,B).

To validate the modeled noise against the noise present on real CT artifacts, we correlated the 2D histograms of simulated artifacts with those of real artifacts (Supplementary Figure 8). Accordingly, we obtained a *“best fit”* simulation for each real case. As expected, low-noise real CT artifacts correlated better with low-noise simulations and vice versa.

**FIGURE 8:**
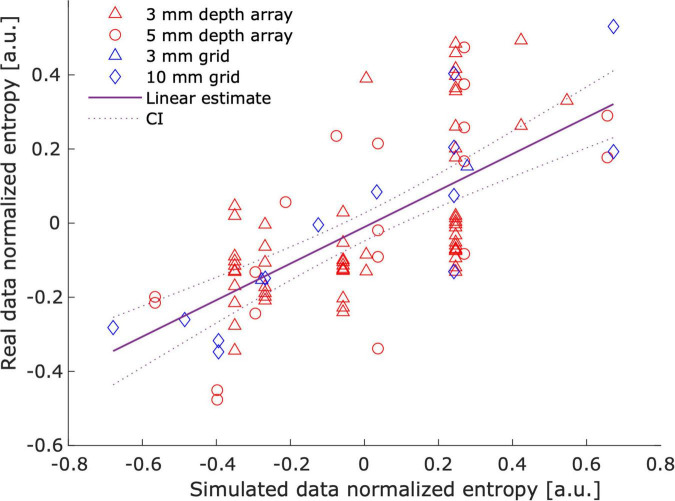
Correlation between real and simulated noise. The figure illustrates that real data noise linearly correlates with simulation noise. The entropy of the cross-correlation between 2D histograms was used as a proxy for noise levels. Individual marks represent a “best fit” simulation – real data pair. The best fit simulation was determined by the highest correlation between their corresponding 2D intensity-radius histograms (see Supplementary Figure 8). Entropy values were normalized (mean subtracted) to account for the effect of artifact size.

To obtain an overall perspective of this relationship, we fitted a linear regression model between the estimated noise level (i.e., entropy) of the *best fit* simulations and the estimated noise level of the real data artifacts. Consequently, the model *real_entropy_normalized* =* β_0_* + *β_1_ simulation_entropy_normalized* was used to assess their relationship. We observed a significant linear correlation between the estimated noise levels of the real CT artifacts and the estimated noise level of their best-fitting simulation counterparts (Figure 8; *β_0_* = −0.010, *β_1_* = 0.493, *R*^2^ = 0.413, *F*(1,92) = 64.409, *p* = 3.187e-12).

### Example I: Evaluation of cluster-based localization methods

To show the usefulness of our simulation framework, we evaluated two electrode localization algorithms in ∼3,300 grid and strip scenarios under 12 different noise level realizations each (∼40,000 simulations in total). Coordinates were localized using *k*-means and *k*-medoids methods and contrasted with the ground truth simulated coordinates to evaluate their accuracy (Supplementary Figure 9 shows two exemplary results).

**FIGURE 9:**
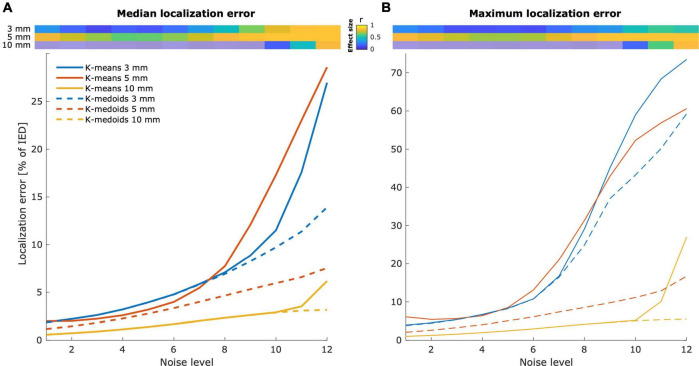
Localization error is affected by the noise level. Localization errors from *k*-means and *k*-medoids clustering algorithms. Localized coordinates were contrasted with the ground truth simulated coordinates to obtain localization errors. The median **(A)** and the maximum **(B)** localization error increase with the simulated noise level. Grids and strips of 3 and 5 mm IEDs are more likely to be affected by noise than arrays of 10 mm IED. The top images show the effect size of the difference between *k*-means and *k*-medoids methods. Non-significant differences are depicted with semitransparent colors.

As expected, the median and maximum localization errors were affected by noise levels (Figure 9). *K*-medoids median localization error was below 15% of the IED when considering all noise levels and below 5% for medium and low noise levels. Instead, *K*-means showed larger median and maximum errors in all noise levels for IEDs of 3 and 5 mm arrays, and for the three highest noise levels in 10 mm IED arrays (Wilcoxon signed-rank test, *p* < 0.05, FDR corrected).

Large effects (*r* > 0.5, Cohen, 1992) in the median localization error were observed across all noise levels in 5 mm IED grids. Instead, 3 and 10 mm IED arrays showed large effects for the largest three and the largest noise level, respectively (Figure 9A, top). Similar effects were observed for the maximum localization error (Figure 9B, top).

### Example II: Interactive simulations in patient’s native brain space

To further demonstrate the usefulness of our platform, we tested the algorithms on a patient’s native anatomical brain space (i.e., unnormalized space). We successfully simulated grids, strips, and depth electrodes using the dedicated controls in the GUI. The interactive interface allowed us to define their locations and orientations precisely. The 2D and 3D views provided a clear interpretation of the array locations in relation to the brain’s pial surface and subcortical structures. Moreover, observing the subject-specific parcellation atlas aided in defining their anatomical location.

**FIGURE 10:**
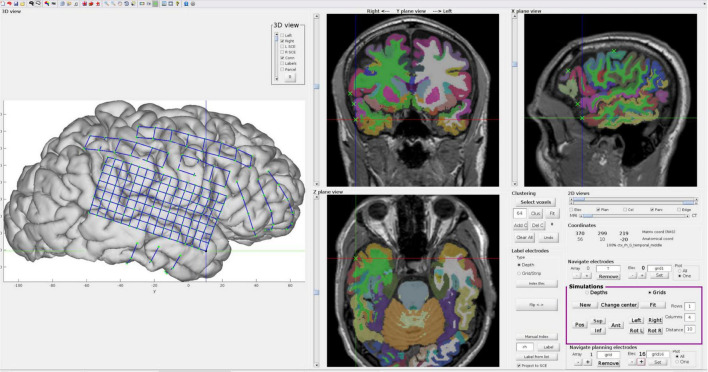
Interactive electrode simulations on a patient’s native brain anatomy. Example of electrodes simulated on a native space brain anatomy using the interactive GUI provided in iElectrodes. The controls (magenta box) are used for displacing and rotating 2D flat grid or strip models over the SCE surface (similar to Figure 2D). The 3D view is particularly useful for defining the location of the arrays in relation to the lateral pial surfaces, whereas the 2D views help to identify their relation to sulci and deep structures. When the desired location and orientation are achieved, arrays are fitted to the SCE surface. For depth electrodes, target and entry points are manually defined in the 2D views. The 2D slices show the Destrieux atlas (color coded), the T1-weighted MRI image, and the simulated coordinates (green ‘*x*’), making the anatomical identification of the arrays easier. Note that electrodes were fitted to the SCE surface, but the pial brain surface is shown for illustrative purposes. SCE, smooth cortical envelope. GUI, graphical user interface.

Figure 10 shows multiple electrode arrays simulated over the pial surface of a single patient. A high-density grid (5 mm IED) was simulated over the right fronto-temporo-parietal region, and its fifth row was particularly aligned with the superior temporal gyrus. Smaller grids were simulated over the fronto-parietal cortex, and strips were simulated to cover the anterior lateral frontal and orbitofrontal cortices, and the middle and inferior temporal gyrus. Note that grid electrodes were not forced to contact the pial- but the SCE surface.

## Discussion

### Summary of results

Several automatic or semi-automatic electrode localization algorithms have been developed in recent years (Arnulfo et al., 2015; Hamilton et al., 2017; Branco et al., 2018a,b; Granados et al., 2018; Centracchio et al., 2021). However, given the lack of ground truth data, the validation of these methods lacks standardization, imposing a major limitation on one of the most important benefits of analyzing brain activity from intracranial recordings, namely its excellent spatial resolution. Therefore, how can we compare the precision of different localization methods validated using different, usually small, datasets? Moreover, how do we know that these datasets represent important features (e.g., noise) of the data we are analyzing in a particular study? These questions are not easily answered, but we expect that simulations can provide a closer approximation to the ground truth in the attempt to answer them.

We provide the first modeling platform of intracranial electrodes to establish a test bench for electrode localization algorithms. Our simulations covered a wide range of realistic scenarios that can be useful for testing localization algorithms. To achieve our aim, we developed novel methods to model intracranial EEG electrode coordinates and the CT artifacts typically produced by these. We mimicked realistic scenarios by simulating implanted arrays of various geometrical dimensions in a wide range of cortical curvatures. We simulated grid and strip coordinates by fitting models to the SCE surface. In addition, we modeled depth electrodes between anatomical target and entry points and precisely defined their bending profile.

Then, we simulated the CT artifacts at the electrodes’ coordinates. The signal intensity distribution over space, the shape of the artifacts, and the orientation of each cluster of high-intensity voxels were carefully modeled. Moreover, different noise levels were simulated and validated with real data.

An extensive database with ∼50,000 cases is freely available for this purpose (see Data availability statement). We focused on providing control over those situations where localization algorithms might fail or encounter difficulties, such as high-density arrays (grids and depth electrodes), high noise levels, overlapping grids, or highly curved implants.

Finally, we showed the impact of noise on the accuracy of two electrode localization algorithms.

### Modeling implantation coordinates

From a visual perspective, simulated coordinates in grids, strips, and depth electrode arrays resemble those of real data. Grid and strips naturally followed the SCE curvature with almost uniformly distributed coordinates, whereas depth electrodes depicted realistically curved trajectories. Moreover, we modeled overlapping CT artifacts for grids and strips, controlling the size, orientation, and the number of overlapping electrodes. These features add realism to the models not shown before.

To objectively evaluate the accuracy of grid and strip implantation models, we measured their deformation (i.e., changes in the inter-electrode distance). Fitting grids or strips over the SCE surface introduce deformations since plane- or line-shaped models must bend to fit the curved brain envelope surface. Importantly, deformation values were relatively small (<0.3% of the IED), indicating that the resulting models are reliable. As an upper bound reference for our method, we measured the IED deformations in real arrays. These deformations were one order of magnitude larger, indicating that our method did not introduce unrealistically high deformations. In our approach, we discarded simulations where the individual IED deformations exceeded 5% of the IED, which might be overly conservative.

As shown in Figure 5A, grid deformations increased with larger IEDs. We theorized that bigger grids had to deform more to cover a larger extent of the curved SCE surface, in contrast to smaller grids that suffer less deformation in the reduced area they overlay. Similarly, higher levels of curvature produced larger deformation values (Figure 5B).

Parameter *K* (Equation 1) controls the deformation introduced in the fitting procedure. We defined *K* = 1,000, which allowed very small deformations (Trotta et al., 2018). However, for large high-density grids (IED of 3 mm), we had to reduce the value to *K* = 100 since the algorithm did not reach feasible solutions in most scenarios. We suggest using the highest *K* value that reaches a feasible solution, therefore introducing the smallest deformations.

Grid and strip electrodes fitting to the SCE were constrained by ε, a tolerance distance between these two. Overall, the resulting distances between electrodes and SCE were negligible compared to the IEDs, indicating successful algorithm performance.

Depth electrode arrays, on the other hand, bend during implantation, mainly due to brain shift.

Our simulations successfully resulted in deformation values equivalent to those observed in real data (Supplementary Figure 3A) and those reported in clinical practice (∼1–3 mm at the tip; Cardinale et al., 2013; Vakharia et al., 2017; Granados et al., 2018). Interestingly, the bending pattern was not symmetrical as expected, and further research is needed to understand its behavior.

Our approach provides precise bending control by applying an arc-shaped function that departs from the original straight linear model. We used a symmetric Lanczos window function for this purpose. However, the use of other arc-shaped functions is straightforward.

Our approach does not consider deviations from the planned implantation trajectory, which can introduce other errors (Vakharia et al., 2017; Granados et al., 2018).

Besides the simulations on MNI space, we also showed the feasibility of simulating on individual (real) subject space. This adds to building even more realistic simulations that could help map expected localization errors at the individual level.

### Simulation of computer tomography artifacts, noise, and overlapping electrodes

CT artifacts corresponding to grids and strip electrodes were placed over the SCE surface, keeping the electrode’s principal axes orthogonal to the surface. Meanwhile, the ones corresponding to depth electrode arrays penetrating the brain were aligned to the arrays’ main axes (Figure 6). From a visual perspective, simulated artifacts echo those observed in real data.

Previously, models of individual electrode CT artifacts were done as uniform intensity cylinders without considering the details examined in the current study (Brang et al., 2016). We defined ellipsoid-shaped artifacts, with the intensity changing as a function of the radius. The electrode models produced a histogram profile resembling real artifacts, i.e., an intensity decrease with increasing radius (Figure 7).

The introduction of noise played a substantial role in producing realistic CT artifacts and was captured by the intensity-radius histograms (Figures 6, 7). Adding correlated noise introduced realistic shape deformations and was visually perceived to affect the center of mass of the electrodes. This feature provides a wider range of difficult scenarios and makes localization more challenging.

A classical approach for characterizing noise in medical images is the subtraction of a smoothed mean image. However, this approach is inappropriate for intensity transitions like those present in intracranial electrode artifacts (Gravel et al., 2004). To circumvent this limitation, we used an information theory approach inspired by techniques quantifying image focus (Bove, 1993; Kristan and Pernu, 2004). In this respect, the measure’s sensitivity to simulated noise was an important consistency check (Supplementary Figures 6, 7), which allowed us to show that noise levels in simulated artifacts mimic those observed in real data (Figure 8). Note that our model explained ∼40% of the real noise variance, indicating their high similarity.

### The relevance of intracranial electrode models for localization algorithms

Over the last decade, several approaches have been proposed to localize intracranial electrodes based on CT and MRI images. The majority used post-implantation CT and pre-implantation MRI images.

The detection of CT artifacts has typically been a manual process (Princich et al., 2013), but has recently been approached by semiautomatic techniques such as clustering voxels of high intensity (Taimouri et al., 2014; Blenkmann et al., 2015, 2017; Brang et al., 2016; Qin et al., 2017; Branco et al., 2018a; Granados et al., 2018), shape analysis (Centracchio et al., 2021), or the interpolation of coordinates given entry and target points in depth electrodes (Arnulfo et al., 2015; Li et al., 2020). Moreover, several approaches have been integrated in novel processing pipelines (LaPlante et al., 2016; Blenkmann et al., 2017; Groppe et al., 2017; Stolk et al., 2018; Li et al., 2020; Davis et al., 2021; Rockhill et al., 2022), providing users several alternatives and even handling group studies (Deman et al., 2018).

Noise and overlapping electrodes are two well-known difficulties for localization algorithms, which hamper the success of automatic methods. For example, Brang et al. (2016) excluded overlapping electrodes from the analysis, given the resulting difficulties, while others treated such cases manually (Taimouri et al., 2014; LaPlante et al., 2016; Branco et al., 2018a; Centracchio et al., 2021). In the same vein, Narizzano et al. (2017) observed errors in their depth electrode estimations associated with other electrodes in close proximity, requiring manual intervention from the user. Moreover, noise signals could be mistakenly detected as electrodes (LaPlante et al., 2016), whereas CT image resolution affects localization accuracy (Brang et al., 2016). Apart from the studies above, the effect of SNR on electrode localization algorithms’ accuracy was rarely discussed, most likely due to the lack of standardized measures to quantify the noise level. The framework proposed here provides a controlled simulation of noise levels and overlapping electrodes, allowing performance evaluation of localization algorithms.

Recently, Centracchio et al. (2021) proposed a novel approach to detect electrode artifacts using a Gaussian support vector machine. Classification accuracy was very high in the analyzed datasets. However, these were restricted to a small number of geometries and electrode sizes. Similar results were obtained by applying deep learning to depth SEEG and DBS electrodes (Vlasov et al., 2021). Approaches like these could strongly benefit from large databases, such as the ones offered here, making their results generalizable across a wider range of geometries.

The spatial resolution of grids and depth electrodes has increased over the last years (Chang, 2015; Erhardt et al., 2020), and high-density arrays are more informative than low-density ones in both cognitive (Gupta et al., 2014; Jiang et al., 2018) and clinical research (Stead et al., 2010). High-density electrodes require additional spatial precision and can be an obstacle for many of the frequently used localization algorithms (Hamilton et al., 2017; Narizzano et al., 2017; Branco et al., 2018b). Simulations can be a reliable platform for developing novel localization techniques for high-density electrode arrays.

Importantly, our models introduced deformations on the order of 1/1,000 of the inter-electrode distance, and distances between electrodes and the SCE on the order of 1/100 mm, which ensure precise modeling of the grids and strips. These errors and deformations were negligible compared to those observed with previous localization (∼0.2–0.6 mm; Blenkmann et al., 2017; Narizzano et al., 2017) and brain-shift correction algorithms (∼2–3 mm; Brang et al., 2016; Branco et al., 2018a,b; Trotta et al., 2018).

As a proof of concept, we evaluated two electrode localization algorithms, *k*-means and k-medoids. Knowing the ground truth location allowed us to characterize how noise levels affected their accuracy, and to have strong evidence to conclude that *k*-medoids perform better than *k*-means in most situations. In a similar way, the current framework has been used to develop a novel localization algorithm called *GridFit*, which is embedded in the iElectrodes toolbox^1^ (Blenkmann et al., 2017; more details about the algorithm will be provided in a forthcoming publication). The simulation platform was used to define the optimal set of parameters needed to precisely localize electrodes under controlled levels of noise, overlap, and curvature. The simulated scenarios helped to identify the sensitivity of the parameters to these variables independently. The same achievement would have been impossible using real data, given the scarce nature of the data and the lack of control of the variables of interest. It is our intent that others find the simulation platform useful to develop novel localization tools.

### Assumptions, limitations, and future directions

Although we provide a substantial number of scenarios and a good starting point to model implanted electrodes, there are some limitations in the current models.

First, we took a simple approach to the spatial intensity distribution of CT artifacts. The simplistic assumption allowed us to build realistic models of large arrays of electrodes.

However, more sophisticated approaches could be implemented, considering the x-ray interaction with metallic electrodes (i.e., beam hardening, scatter effects, and Poisson noise), and the corresponding alteration produced in the image reconstructions (Boas and Fleischmann, 2011; Katsura et al., 2018). Developments in this direction could pave the way to model the artifacts produced by microwires at the tip of depth electrodes (e.g., Behnke–Fried electrodes, Ad-Tech Medical) and the design of novel localization algorithms for this specific and unsolved problem.

Moreover, alterations in the electrode shapes might be caused by beam hardening (Boas and Fleischmann, 2011; Centracchio et al., 2021), and in some situations, streak artifacts can be prominent in CT images, but these are typically removed by thresholding (LaViolette et al., 2011b). Accordingly, we modeled electrode artifacts after thresholding, as most algorithms are applied to this type of data. However, there might be some cases where thresholding is a fundamental step to assess (e.g., Davis et al., 2021). CT image augmentation and more complex models need to be developed for such instances.

Second, the implantation of intracranial grids and strips is a procedure that results in brain tissue deformation. Deformations of 10 mm or more can occur on the brain surface around the electrodes or in deeper brain structures due to cerebrospinal fluid loss in the ventricles (Studholme et al., 2001; LaViolette et al., 2011a). Implantation of depth electrodes might also produce brain deformations, but to a much lower extent, with a smaller amount of cerebrospinal fluid loss, if any (Elias et al., 2007). Modeling brain deformations is a challenging problem, where multiple variables have to be considered, such as the size and location of the skull opening, the amount of cerebrospinal fluid loss, and the swelling of soft tissue, among other factors (Studholme et al., 2001). Given the complexity of the problem, we assumed non-deformed brains in our simulations, which precludes their use to evaluate brain-shift correction algorithms. The use of non-linear finite element methods can be a successful way to model these more complex brain deformations (Wittek et al., 2007).

Finally, we should mention that the volume covered by the target coordinates in the depth electrode simulations is far from covering the full brain volume. Similarly, we did not cover the complete lateral cortex with grids and strips. However, this is possible in the current framework by carefully selecting the entry and target coordinates, seed points, electrode sizes, and geometries.

## Conclusion

Intracranial EEG recordings allow us to study brain function with excellent spatial resolution and rely on precisely localizing the implanted electrodes. Here, we presented the first platform to model electrode coordinates and CT artifacts of implanted grids, strips, and depth electrodes.

Implanted electrodes under realistic scenarios were successfully modeled with high accuracy, resembling real cases. These methods enable the systematic and quantitative evaluation of electrode localization strategies, contributing to the development of more accurate techniques. The platform could be a starting point for more sophisticated models, such as brain tissue deformations or microwires.

The modeling methods and simulation results are freely available to the research community via open repositories. Moreover, a graphical user interface implementation is also available via the open-source iElectrodes toolbox.

## Data availability statement

The scripts and simulated datasets presented in this study can be found in online repositories. The name of the repository and accession number can be found below: Center for Open Science (COS) Open Science Framework (OSF), https://osf.io/9fsm3/, doi: 10.17605/OSF.IO/9FSM3. A graphical interface for the interactive simulation of electrode coordinates is available within the iElectrodes toolbox at https://sourceforge.net/projects/ielectrodes/. The patients’ datasets analyzed in this study are not publicly available due to our ethical approval conditions that do not permit public archiving of anonymized study data.

## Ethics statement

The studies involving human participants were reviewed and approved by the Regional Committees for Medical and Health Research Ethics, Region North Norway (REK 2015/175), and the Human Subjects Committees at UCSF, UC Irvine, and UC Berkeley. The patients provided their written informed consent to participate in this study.

## Author contributions

AOB, A-KS, and TE designed this study. AOB coded and tested the software tools, performed the analyses, and wrote the manuscript. TE, A-KS, PL, JI, and RK provided the data and validated the results. All authors revised the manuscript, read, and approved the final manuscript.

## Footnotes

1
https://sourceforge.net/projects/ielectrodes/

## Abbreviations

IED: inter-electrode distance
SCE: smooth cortical envelope
SNR: signal to noise ratio.
