# Supplementary material for: Modeling intracranial electrodes. A simulation platform for the evaluation of localization algorithms
Source: Front Neuroinform. 2022 Oct 6;16:788685. doi: 10.3389/fninf.2022.788685 (PMC9582989; doi:10.3389/fninf.2022.788685)
Supplement: Supplementary file 1 [file Data_Sheet_1.pdf]

Supplementary material

S1. Pial surface and Smooth Cortical Envelope

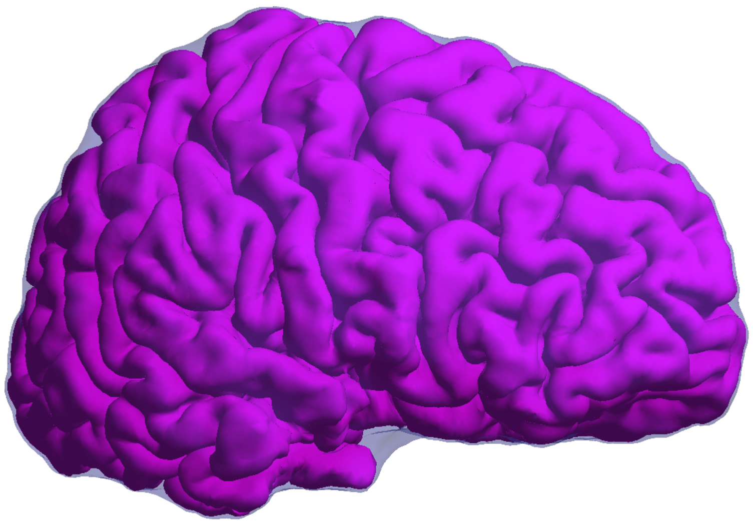

Supplementary Figure 1. Pial surface and Smooth Cortical Envelope.
Smooth Cortical Envelope (SCE, in semi-transparent violet) over the pial surface (in pink) computed for an individual subject's brain. The SCE is enclosing the pial surface using a 30 cm sphere. Simulated grids and strips were overlaid on the SCE surface.
The same procedure was also applied to the MNI standard brain.
SCE: Smooth Cortical Envelope

S2. Selection of seed points for simulations

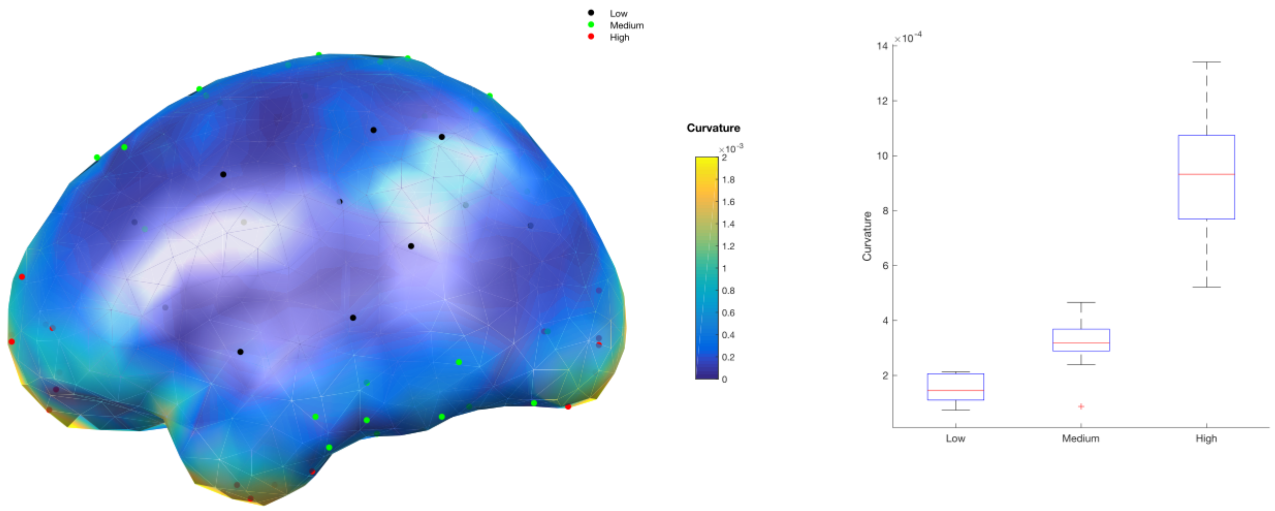

Supplementary Figure 2. Curvature of SCE and seed points.
Left: Local curvature of a smoothed version of the MNI SCE showing the selected seed points in Low (black), Medium (green), and High (red) curvature regions.
Right: Boxplots showing the curvature values for seed points in Low, Medium, and High curvature areas. Curvature values were computed for each seed point as the mean within 25 mm.
The center line (red) of each boxplot represents the median and the edges are the 25th (Q1) and 75th (Q3) percentiles. The whiskers extend to the most extreme data points not considered outliers, and the outliers are plotted individually.
SCE: Smooth Cortical Envelope

S3. Modeling grid and strip overlaps

The following steps are performed to model overlaps:

1. A percentage of overlap is defined a priori (e.g., 10%), which sets the *number of overlapping electrodes* (NOE).
2. To define spatial rotations, a set of reference points are needed. These are the same electrode coordinate points in the case of grids or the SCE surface points in the vicinity of the electrodes (15 mm radius) in the case of strips.
3. Reference points are projected to a principal component (PC) space (Figure 3A), where the third component (PC3) has the lowest variance. In the case of strips, the same transformation is applied to the electrode coordinates.
4. A 2D surface S_{fit} is fitted to the reference points using a local linear regression algorithm (Matlab *fit* function using *'lowess'* option, Figure 3A).
5. Within a 2D space defined by the first two PCs, overlapping grids or strips are defined over the original array and with a given orientation (Figure 3B).
6. The overlapping electrode array is stepwise translated:
    a. The overlapping array is translated (with a step size of IED/100) in a defined outward direction (Figure 3C).
    b. The number of overlapping electrodes in the original electrodes' vicinity is counted in each step (within a distance equal to IED $\sqrt{2}$).
    c. The translation stops when the number of overlapping electrodes reaches the NOE (Figure 3C); otherwise, repeat all prior steps.
7. Electrodes from the overlapping array that are outside the main array area are discarded.
8. The third dimension (PC3) of the overlapping array is computed using the S_{fit} surface.
9. A space between arrays is added to represent their thickness (1 mm in the PC3 dimension).
10. Electrode coordinates are projected back to the original 3D space (Figure 3D).
11. The normal vector at each electrode is computed as the mean of the closest normal vectors from the original array, weighted by their distance.

S4. Deformations in real data

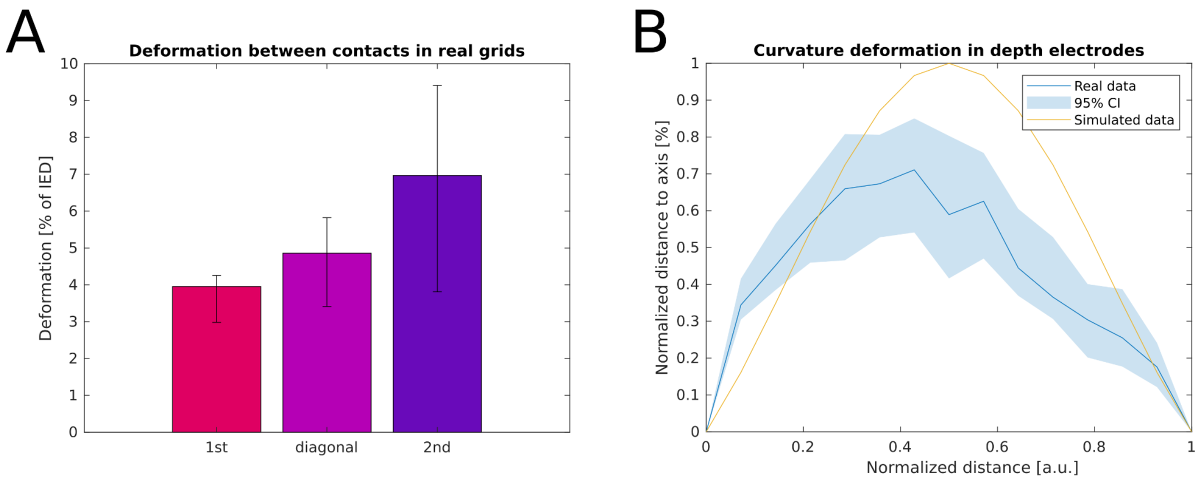

Supplementary Figure 3. Deformation characteristics of real grids and depth electrodes.
A. *Median deformation between 1st, 2nd, and diagonal neighbor contacts in 10 mm real grids. Error bars denote 95% CI of the median. Median values are 3.95, 4.85, and 6.96 [% of IED] for 1st, 2nd and diagonal deformations, respectively.*
B. *Curvature deformation for depth electrodes. The median distance to the main axis of the array was measured for each contact (blue line), and the corresponding 95% CI of the median. The array distance has been normalized between 0 and 1; 0 represents the deepest, and 1 most lateral contact in each array. The yellow line represents the Lanczos window function applied to deform simulated data (1% maximum deformation was used). The average peak deformation for depth electrodes was 0.82 %, 95% CI [0.72, 1.01], comparable to the 1% maximum deformation implemented in our approach, but centered at 0.37, 95% CI [0.33, 0.43]).*

S5. Distance between contacts and SCE in simulated data

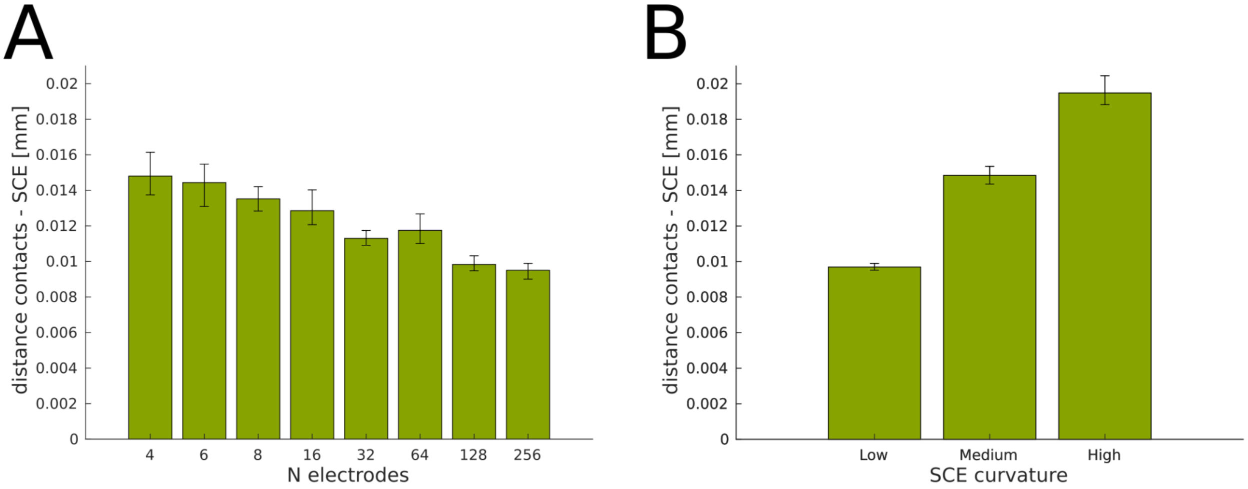

Supplementary Figure 4. Deformation characteristics of real grids.
A: *Median distance between electrodes and SCE as a function of the number of electrodes.*
B: *Median distance between electrodes and SCE as a function of the SCE curvature.*
Error bars denote 95% CI of the median. SCE: Smooth Cortical Envelope. IED: Inter-Electrode Distance.

S6. Entropy of real and simulated electrode arrays

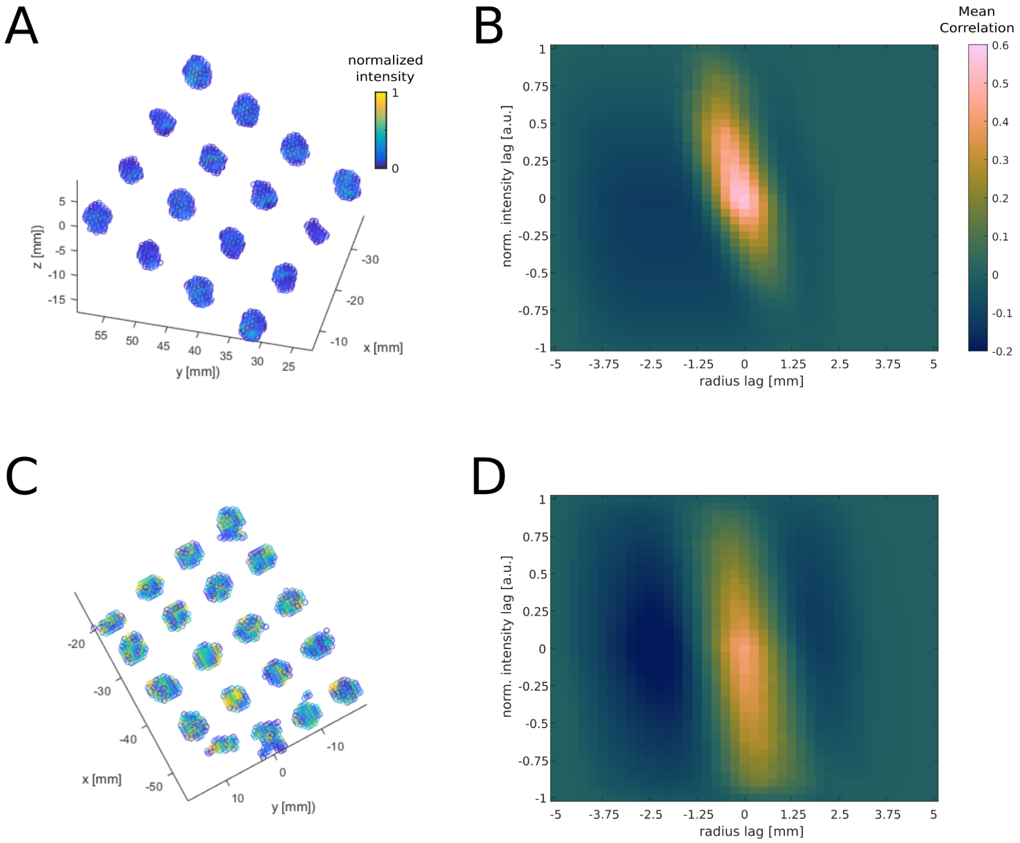

Supplementary Figure 5. Examples of average cross-correlation in real cases
*Plots **B** and **D** show the mean cross-correlation of 2D intensity-radius histograms obtained from relatively low (**A**) and high noise (**C**), 10 mm IED, real grid cases, respectively. Low noise cases usually produced sharper cross-correlation images, e.g., B, whereas higher noise levels were typically associated with smother cross-correlations, e.g., D. The median entropy values associated with these exemplary arrays were 1.42 and 2.17 respectively.*

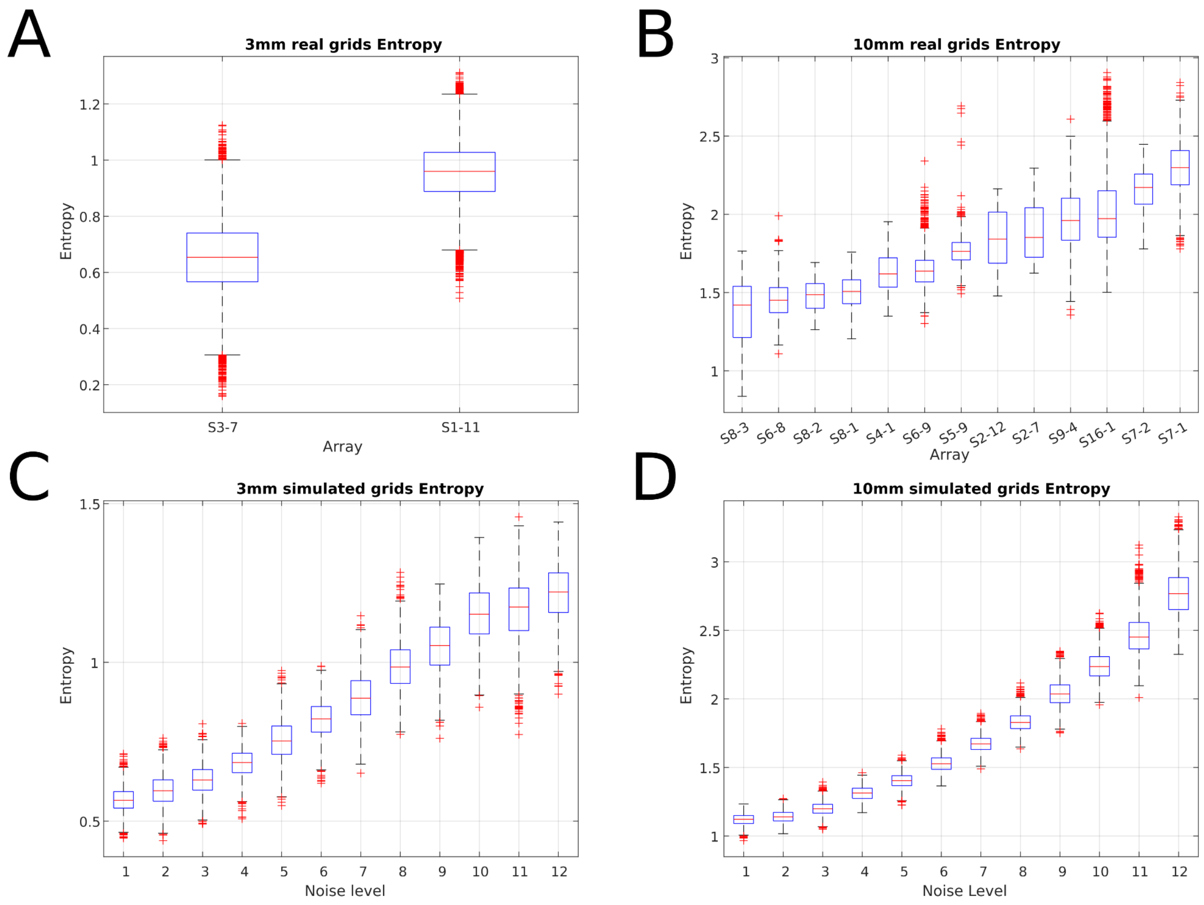

Supplementary Figure 6. Entropy of real and simulated grids.

*Boxplots showing the entropy of 3 mm (**A**) and 10 mm (**B**) real grids. Entropy of 3 mm (**C**) and 10 mm (**D**) simulated grids. Entropy was calculated on the cross-correlation intensity-radius histograms across electrodes (as shown in Sup. Fig 5).*

The middle line of each box depicts the median and the bottom and top of each box are the 25th and 75th percentiles of the sample, respectively. Whiskers go from the end of the interquartile range to the furthest observation within the whisker length. Observations beyond the whisker length are marked as outliers if located more than 1.5 times the interquartile range away from the bottom or top of the box.

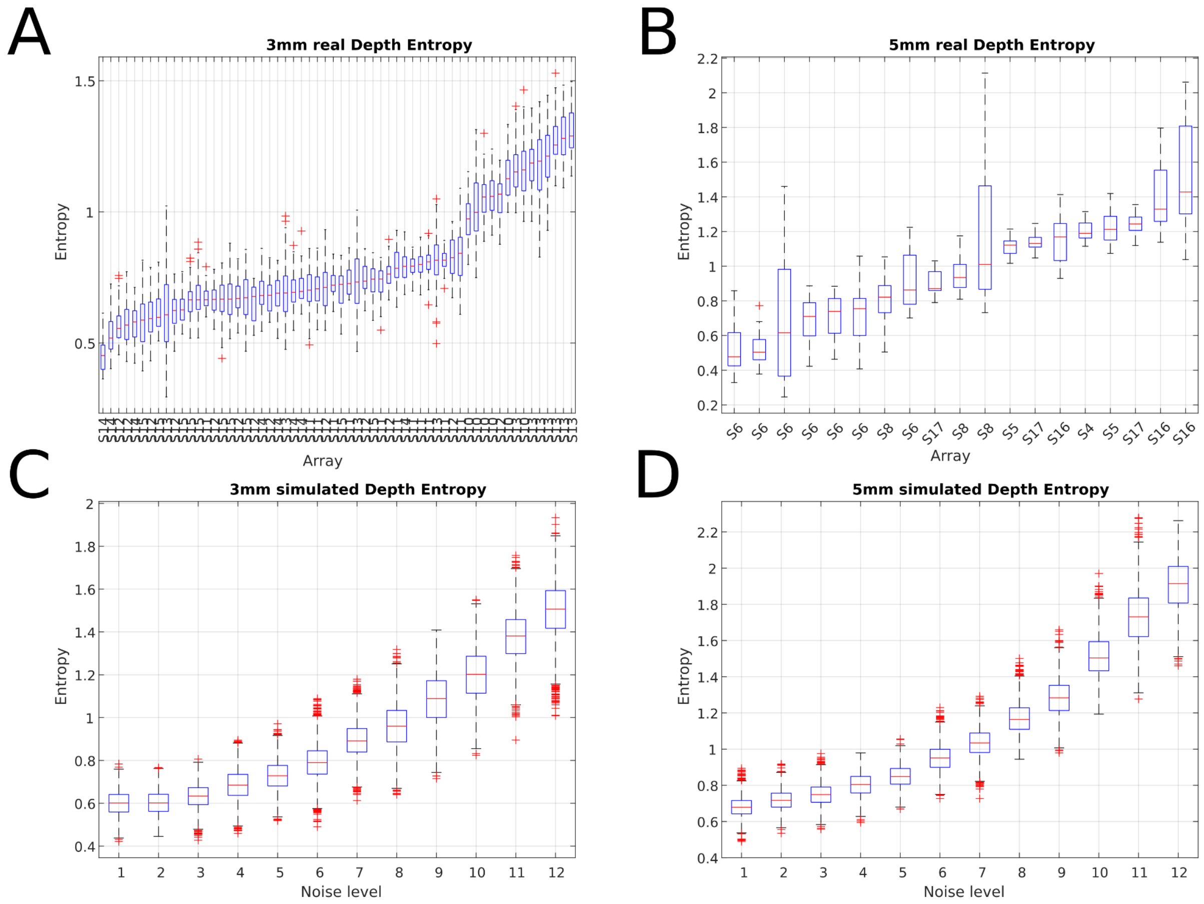

Supplementary Figure 7. Entropy of real and simulated depth electrodes.

*Boxplots showing the entropy of 3 mm (**A**) and 5 mm (**B**) real depth electrodes. Entropy of 3 mm (**C**) and 5 mm (**D**) simulated depth electrodes. Entropy was calculated on the cross-correlation intensity-radius histograms across electrodes (as shown in Sup. Fig 5).*

The middle line of each box depicts the median and the bottom and top of each box are the 25th and 75th percentiles of the sample, respectively. Whiskers go from the end of the interquartile range to the furthest observation within the whisker length. Observations beyond the whisker length are marked as outliers if located more than 1.5 times the interquartile range away from the bottom or top of the box.

S7. Normalized correlation between real and simulated data

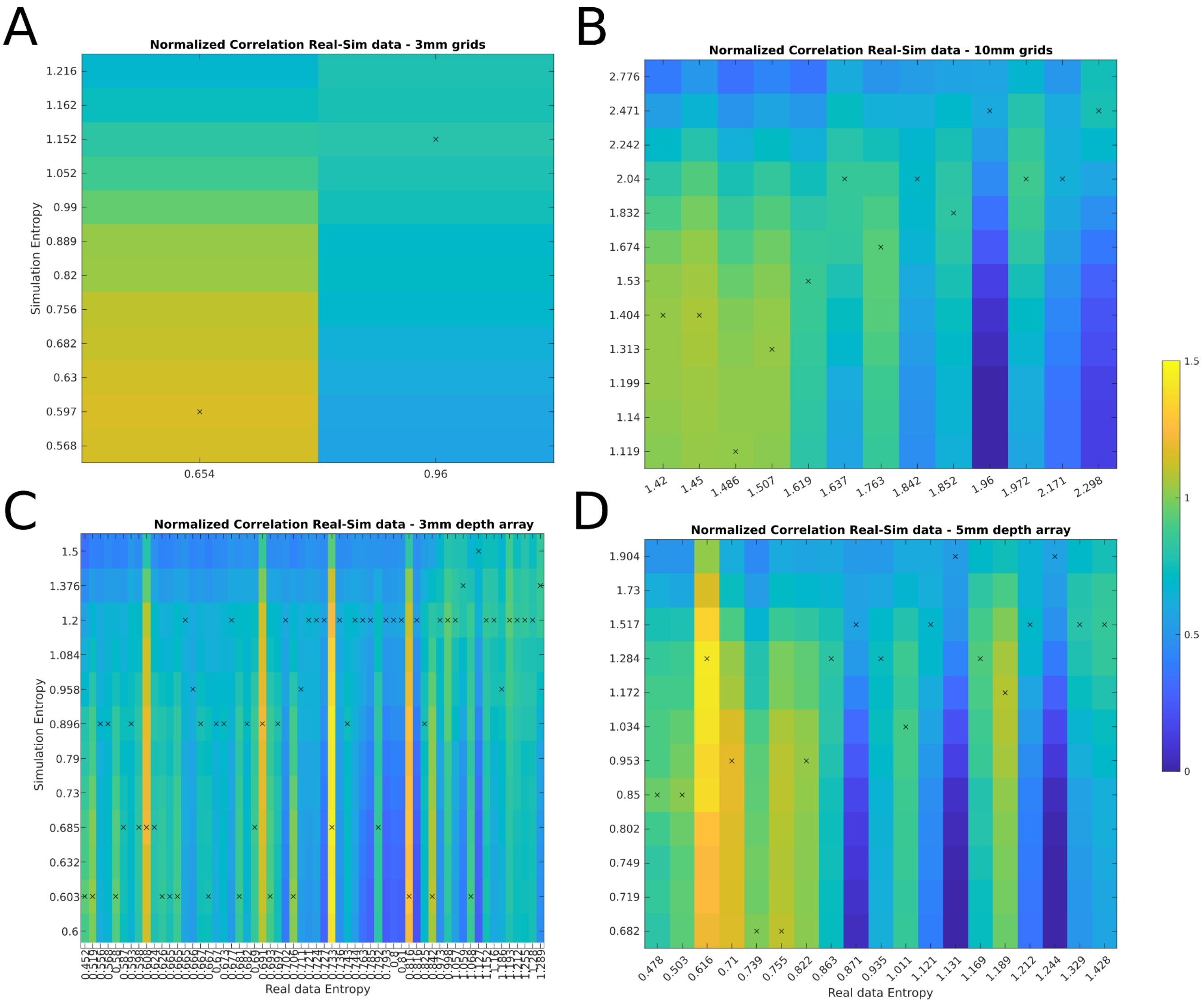

Supplementary Figure 8. Correlation between real and simulated data.
*Normalized correlation was calculated between intensity-radius histograms across electrodes of the real (x-axis) and simulated (y-axis) arrays for 3 mm (**A**) and 10 mm (**B**) grids, and for 3 mm (**C**) and 5 mm (**D**) depth electrode arrays. The color coding indicates the normalized correlation values.*
Crosses indicate the highest correlation (best fit) between simulated and real arrays. These pairs of real-simulated arrays are then used in the linear model depicted in Figure 8.
Data were sorted according to their entropy levels. Entropy was calculated on the cross-correlation intensity-radius histograms across electrodes (Sup. Fig 5). Entropies for each array are shown in Sup. Figures 6 and 7.

S8. Electrode localization example

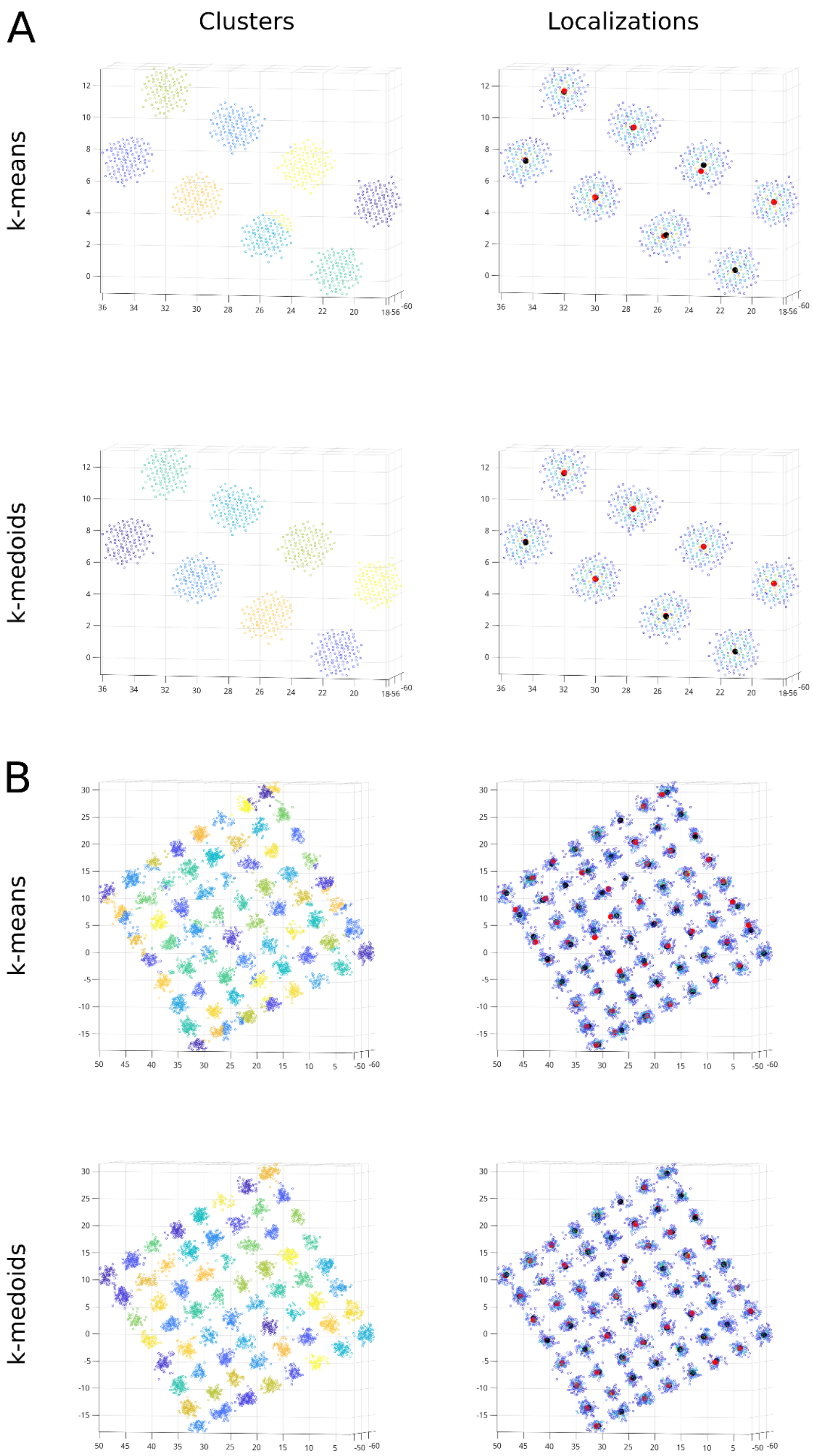

Supplementary Figure 9. Localization examples for k-means and k-medoids algorithms.

3D plots show two examples of CT artifact localization. ***A*** *shows results for a 2 x 4 grid array, 5 mm IED, with added low noise level (level 1), and* ***B*** *for a 8 x 8 grid array, 5 mm IED, with medium-high noise level (level 8).*

For each example, the top row shows the k-means results, and the bottom row shows the k-medoids results. Left plots show the voxels associated with each identified cluster (color-coded). In the right plots, black points denote the true electrode coordinates (simulated), and red points denote the localized ones. Notice that some voxels were assigned to incorrect clusters in the k-means algorithm.
